# Redefining success and finding community: an interview with Atom Lesiak and Zara Weinberg on the importance of building support networks for transgender scientists

**DOI:** 10.1038/s42003-022-03246-7

**Published:** 2022-03-31

## Abstract

As part of our celebration of Transgender Day of Visibility, we asked transgender scientists about their research experiences, role models, and the importance of accountability and diversity in academic environments.

Dr. Atom Lesiak (they/xe/she) is the Director of Education Outreach for Genome Sciences at the University of Washington, where they develop innovative programs to teach science and provide hands-on training in K-12 schools.

**Figure:**
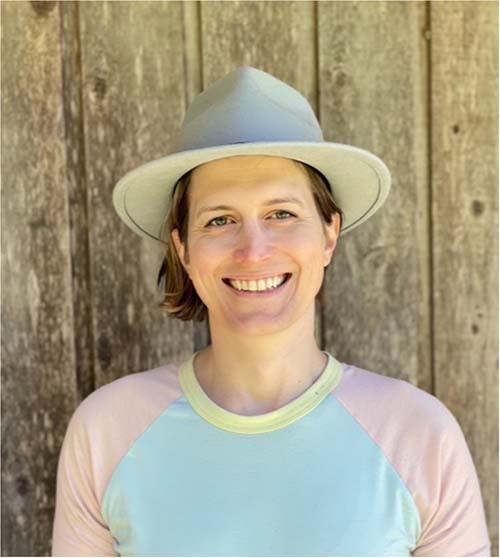
Atom Lesiak.

Dr. Zara Weinberg (she/her) is a postdoctoral fellow at the University of California, San Francisco, where she studies cyclic AMP signaling in different cell types of the early embryo.

**Figure:**
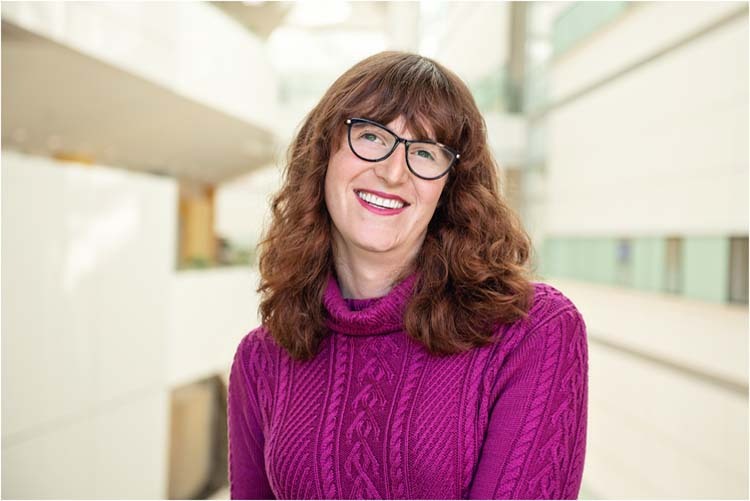
Zara Weinberg.

Please tell us about your academic background and current position.

**Atom Lesiak (AL):** For most of my training I studied the brain. I started out studying the brainwaves and brain development of neurologically diverse children, transitioned to studying the genetic and molecular mechanisms of the brain as a graduate student and postdoc. My goal has always been to better understand the brain in order to develop better therapeutics for anxiety and depression. I unofficially “minored” in studying brain gender on the side to try and better understand the phenomenon of my own gender expansive identity. As a postdoc at the University of Washington I volunteered my time towards science outreach (public lectures and exhibit facilitation) while also helping run a transgender support group. Now, as the Director of Education Outreach at Genome Sciences at the University of Washington, I get to integrate my volunteer and research work to help support Diversity, Equity and Inclusion initiatives at my institution and develop science education curriculum and activities for public education.

**Zara Weinberg (ZW):** When I started my undergraduate education at Reed College, I was determined to major in linguistics. By the end of my third semester, I realized that although I found language fascinating, what truly fascinated me was the way the brain constructed it. At the time, Reed had no neuroscience major so I worked with my adviser Dr. Paul Currie to major in Psychology while taking as many biology and chemistry courses as I could. Working with Paul, I was able to participate in several research projects, where I led my own explorations into the role of the feeding hormone ghrelin in motivation and reward. Through my research, I became fascinated with cellular receptors and the ways that multiple drugs can act the same receptor to produce distinct effects.

When I began my PhD at Carnegie Mellon University, my fascination with receptors guided me to the lab of Dr. Manojkumar Puthenveedu, who studied how receptor movement within cells played an essential role in receptor signaling. Working in Manoj’s lab, I discovered an important aspect of how opioid hormones and opioid drugs differentially signal at the μ opioid receptor. This work, along with many collaborative projects, established my cell biology background with expertise in protein trafficking, cell signaling, and computational image analysis. For my postdoc, I wanted to move beyond single receptors and study the complexity of cell signaling from the perspective of convergent signals that are shared between multiple signaling pathways. I came to the University of California, San Francisco (UCSF) to work with Dr. Hana El-Samad, hoping to gain skills in systems biology and modeling. In the lab, I sought to understand how the second messenger cyclic AMP (cAMP) was able to confer different cellular behavior in different cell types. I am taking a developmental biology approach, investigating cAMP signaling in different cell types of the early embryo. Just as I was beginning this work in earnest, the COVID pandemic rapidly spread and changed my focus dramatically. With encouragement from my mentor Hana, I applied synthetic biology and cell engineering tools from the lab to research questions on SARS-CoV-2. In collaboration with Dr. David Baker’s laboratory at University of Washington, I built engineered cells that could detect and respond to SARS-CoV-2 in a customized manner. I am now continuing my project on cAMP, but I am working to bring aspects of my new cellular engineering expertise into the project. Stay tuned for more on that!

Who has been a role model or key mentor in STEM (LGBTQIA+ or otherwise) that has had an impact on your career?

**AL:** I struggled with authentic self-expression throughout my youth and into my science training. My gender journey has largely paralleled my career journey. I learned the “rules” early and understood the opportunities made available to me by conforming to the expectations of my assigned gender at birth socially and academically. Despite my best efforts to conform, I was unable to completely hide my multi-dimensional eccentricities. It was a struggle to find my gender identity as neither man nor woman (genderqueer), much as it has been to find my identity as a non-traditional research/science outreach academic. I am deeply appreciative of the support my parents and science mentors gave me during my development, while recognizing that they did not have access to the information and tools to fully support or understand me. I knew of successful transgender STEM professionals such as Ben Barres and Lynn Conway, but still couldn’t fully see myself represented in their gender identities or career paths. My greatest mentors came from non-traditional sources. For example, the career counselor that looked at my resume and said I’m applying to all the wrong types of academic positions while I was continually failing to get interviews for faculty positions. I was fortunate to find mentorship among my peers at the transgender support group who taught me invaluable lessons on interpersonal skills, communication, and authenticity. I learned essential skills outside the lab from my teaching mentor and science communication mentors, all the while I was worried that my research mentors would be upset that I wasn’t working in the lab enough. Unfortunately, I needed to pursue this expansive approach to my education independently, finding non-traditional mentorship in my non-traditional career in academia.

**ZW:** I have been incredibly lucky to have many role models and mentors at every stage of my scientific career. I was particularly blessed to be mentored by my PhD labmates, Drs. Mana Zajac and Shanna Bowman. Mana and Shanna guided me through my PhD, supported me, and taught me a lot about what it means to be a mentor and how to support the goals of one’s mentees. As a postdoc, my current mentor Dr. Hana El-Samad has been a true inspiration. Hana has unequivocally supported me, and in many ways sponsored my career trajectory through providing me ample opportunities to shine. Furthermore, Hana’s advocacy for women in science and anti-racism in scientific funding has been a consistent inspiration to me on how to make change in the unyielding world of academic science.

In addition to these mentors, many of my role models in science are my peers and mentees. I’m in awe of the quality of science conducted by my labmates Drs. Steph Crilly and Lindsey Osimiri. I am constantly inspired by my former mentees Candilliane Serrano Zayas and Josh Lott for their ability to navigate through nonsense in order to achieve their goals. I have learned a great deal about advocacy and improving equity from my colleague Roberto Efráin Díaz, UCSF Assistant Dean of Diversity and Learner Success Dr. D’Anne Duncan, and former UCSF Assistant Dean of Postdocs Dr. Gabriela Monsalve. I’m in absolute awe of my contemporaries Dr. Eartha Mae Guthman, Dr. Simón(e) Sun, and Krisha Aghi, as they push the boundaries of the field of neuroendocrinology and work to redefine what it means to study sex as a biological variable.

I have been so lucky to have so many spectacular mentors and to be surrounded by so many wonderful role models. We all could benefit from recognizing the greatness in our peers and look to them for inspiration!

How do you think STEM could better promote inclusive research environments and support transgender scientists?

**AL:** I believe that hubris is the biggest challenge towards diversity, equity, and inclusion in academia. There’s an extraordinary amount of confirmation bias in the academic system, wherein those that have found “success” believe that their road to success is THE road to success. I was taught how to be “successfully” cisgender from “successfully” cisgender people, in the same way I learned how to become a “successful” researcher in academia from “successful” researchers in academia. In both cases “successful” people that cared about me were worried I wouldn’t find success without conformity. I tried and failed to be cisgender in the same way I tried and failed to be a “successful” basic science researcher. Hubris in academia prevents the development of a more expansive and inclusive definition of success, much like gendered bathrooms uphold a rigid expectation of gender expression. To non-conform is to risk being forcibly expelled and un-welcomed. The rigid expectation of “success” and conformity reinforces the hostile and inequitable training environment for diverse individuals. I believe that to create a more inclusive science training environment, we need to re-define “success”, and structure this new more diverse definition of “success” to include equity and sustainability practices. My academic “success”, like my gender “success” was only found through finding my individual authenticity. I feel “successful”, but still struggle to feel safe and welcome. In academia, I felt “successful” when I got my faculty job (officially Lecturer part-time); however, I find myself in a never-ending struggle to support my salary through a hyper-competitive grant system. The grant review system is sustained by those that found “success” within it, much in the same way that gendered bathrooms are sustained by and inclusive of “successfully” cisgender (or cis-passing) people. I continue to struggle to feel safe in expressing myself authentically within academic spaces because often my authenticity challenges the norms and expectations of the system on multiple levels. I want to work towards building a more sustainable and equitable environment in academia to allow for more diverse representation in the system, I just hope I can stick around long enough to do it.

**ZW:** I came out as trans in the middle of my PhD. My labmates and PI were largely supportive, but I was subjected to unprofessional behavior from others at my institution. Administrators demanded that I explain “my situation” to them. Colleagues and professors continued to misgender me years after I came out. Throughout these difficult interactions, I tried to be accommodating of others instead of demanding the respect that I deserve. Some of my trans colleagues chose instead to confront the transphobia and toxicity they experienced, and in some cases, left academic science when their departments refused to address the ongoing harassment. Trans scientists would benefit from academic environments holding their employees accountable for their bigoted attitudes.

But the experience of trans scientists is dependent on more than just our colleagues’ behavior. Many issues faced by academic scientists are amplified for trans people, and worse still for folks who also hold other intersecting marginalized identities, including those who are racialized. According to researchers at the UCLA School of Law Williams Institute, the poverty rate amongst all trans people in the US is almost twice that of cis straight people, and even higher for Black and Hispanic trans people. This means that the suppressed wages in academia disproportionately hurt trans people, and especially trans people of color. Multiple recent studies suggest that all trans individuals are subjected to greater psychosocial burdens than cis people, suggesting that the mental health crisis within academia disproportionately affects trans scientists. Trans people have to overcome significant barriers to access healthcare, an already notoriously difficult process for graduate students and postdocs. Improvements in wages, mental health support, access to and quality of healthcare, and accountability for transphobia are required to make research environments inclusive and supportive of trans scientists.

Additionally, trans people around the world are being directly targeted by legislators that seek to deny us access to healthcare or in some regions, the right to exist. The introduction of anti-trans legislation in 34 states in the US is government-sanctioned eugenics against trans people. As with many issues related to marginalized people, part of making science an inclusive and supportive place is making the culture that scientific institutions exist in a welcoming and supportive place. This is particularly important for researchers studying sex, whose research can be weaponized against trans people. As scientists, we have a responsibility to conduct our research in a socially responsible way, and right now trans people need scientists to re-evaluate how they talk about and study sex. For many trans people, how scientists discuss sex is quite literally a life or death matter.

Trans scientists benefit from the same interventions that many marginalized people at research institutions have been advocating for for years. If you want to improve your institution’s climate for trans people, you need to fight trans rights in your local community and beyond.

What advice would you give to other early-career researchers struggling with their identity?

**ZW:** Any identity that puts you outside the dominant power structure is one of your biggest strengths. For a discipline where we ostensibly value innovation, novel viewpoints, and creative problem solving, science is shockingly conservative in so many ways. By embracing our identities, we are fundamentally bringing a new perspective to our work. Being trans is an important part of my scientific perspective. It helps me question dogma and see beyond false binaries that are ingrained in science. I spent a lot of time worrying that being trans would somehow subsume the rest of my identity—that I would only ever be known as that trans scientist—so for several years after I first came out, I tried to hide it. But being proudly trans has instead enriched my other identities. It has now become another part of the rich tapestry of my life and identity that helps to make me be the best scientist I can be. It can be so scary to come out, to advertise your uniqueness to the world. But in the end it is the only way to be the best version of ourselves.

In addition to seeking the best version of yourself, you should seek community. We are lucky to live in a time where there are robust communities within science for people with a variety of identities. I cried tears of joy after I attended a journal club run by and for trans scholars. It took me until the third year of my postdoc to find my community - don’t wait that long. Find your community now, I promise you won’t regret it.

*Interviews were conducted by Associate Editor George Inglis*.

